# School Violence Exposure as an Adverse Childhood Experience: Protocol for a Nationwide Study of Secondary Public Schools

**DOI:** 10.2196/56249

**Published:** 2024-08-28

**Authors:** Sonali Rajan, Navjot Buttar, Zahra Ladhani, Jennifer Caruso, John P. Allegrante, Charles Branas

**Affiliations:** 1 Department of Health Studies & Applied Educational Psychology Teachers College Columbia University New York, NY United States; 2 Department of Epidemiology Mailman School of Public Health Columbia University New York, NY United States

**Keywords:** adolescents, adverse childhood experiences, gun violence, health outcomes, injury prevention, school violence

## Abstract

**Background:**

Poor mental health and adverse childhood experiences (ACEs) predict extensive adverse outcomes in youth, including increases in long-term risk for chronic disease and injury, impaired emotional development, and poor academic outcomes. Exposure to school violence, specifically intentional gun violence, is an increasingly prevalent ACE. The anticipation of school shootings has led to the implementation of school safety and security interventions that may increase anxiety, depression, and other indicators of poor mental well-being among students and staff alike. Despite this, the association between exposure to existing school safety interventions and early adolescent student mental health outcomes, while accounting for one's history of ACEs, has not been previously investigated.

**Objective:**

The study protocol described here aims to determine whether there is a significant difference in the prevalence of mental health outcomes, perceived school safety, and academic engagement between adolescent students (grades 6-12) at schools who have experienced a school shooting and those who have not; whether existing interventions to promote school safety and security are associated with poor mental health outcomes among students and school staff; and what the strength of the association between school safety interventions and mental health outcomes among students and teachers is in schools that have experienced a school shooting versus schools that have never experienced a school shooting.

**Methods:**

This observational study will collect cross-sectional survey data from a nationwide sample of students, teachers, and principals at 12 secondary public schools across the United States. The participants come from 6 randomly selected exposure schools that have either experienced a recent (<2 years ago) intentional school shooting or have experienced an intentional school shooting less recently (>2 years ago). Data from these schools are being directly compared with 6 secondary schools that have never experienced a school shooting.

**Results:**

Institutional review board approval for this research project was obtained and the study subsequently began its recruitment and data collection phase in January 2024. Data collection is currently ongoing and the expected completion date is January 2025. The analytic plan is designed to determine if the strength of the association between school safety interventions and mental health outcomes differs among students and school staff in schools with varying levels of school violence exposure. Analyses will be used to evaluate the role of ACEs on the relationships among exposure to an intentional school shooting, exposure to school safety strategies, and student outcomes (ie, mental health and well-being, perceptions of school safety, and educational outcomes).

**Conclusions:**

The results from this study promise to generate meaningful and novel findings on the extent to which having a prior history of ACEs moderates the relationships among exposure to intentional school gun violence, school safety strategies, and student outcomes (ie, mental health and well-being, and perceptions of school safety).

**Trial Registration:**

ClinicalTrials.gov NCT06153316; https://clinicaltrials.gov/study/NCT06153316

**International Registered Report Identifier (IRRID):**

DERR1-10.2196/56249

## Introduction

### Background

Poor mental health and the prevalence of adverse childhood experiences (ACEs) have substantially increased among the youth in the United States over the past decade. This increase has been confirmed through independent national surveys, hospital admissions related to self-harm, and death by suicide, which has tripled for girls and doubled for boys aged 10-14 years [1-5]. Moreover, recent data from the Adolescent Behaviors and Experiences Survey conducted by the Centers for Disease Control and Prevention and others [6] have underscored that indicators of poor mental health among adolescent youth have persisted during the COVID-19 pandemic [7]. These increases have occurred concomitant with substantial increases in school shootings across the United States [8,9].

Exposure to gun violence over the past several years has catalyzed a national movement that has sought to increase school gun violence preparedness and safety. K-12 (the grades of kindergarten and 1st-12th grade) schools that have experienced intentional gun violence have implemented a number of policies specifying tactics designed to keep their schools safe and secure from future acts of violence. These have included arming teachers with firearms, using metal detectors, and implementing “zero tolerance” discipline policies [10-14]. Nearly all public schools that have not experienced intentional gun violence have responded to the anticipation of such incidents by implementing some form of school safety and security measures; as of 2019, 96% of all K-12 public schools in the United States reportedly conduct lockdown drills [15].

Although the pursuit of these efforts has value, research suggests that some of these interventions may not be beneficial and may decrease student perceptions of safety in their schools [10-12]. Additional research has drawn connections between perceived school safety and self-reported mental health symptoms among the youth [16]. However, little research has documented the burden of mental health problems on cohorts of students subsequent to actual as well as anticipated school gun violence. Understanding the nature and scope of this burden is critical for designing effective school safety interventions that attend to youth mental health needs while also accounting for prior histories of ACEs among students. In addition, there is scant research evaluating the mental health needs and perceptions of safety among school staff who have been present during a school shooting or who are also exposed to school safety interventions, or both.

### Mental Health and Gun Violence

Poor mental health symptoms among youth predict extensive co-occurring adverse outcomes, including increases in long-term risk for chronic disease and injury, impaired child development, and poor academic outcomes [17-21]. This observed increase in prevalence of mental health problems followed the 2012 Sandy Hook Elementary School shooting, and perhaps not by coincidence—since January 2012, there have been hundreds of incidents of intentional gun violence on school grounds [8,9]. Thousands of children have been exposed to gun violence, specifically in school, during this same period [22]. Indeed, children are now often referred to as being a part of the “mass shooting generation,” a cohort of youth growing up in the presence of gun violence [23].

The prominence of gun violence in communities across the United States is likely and, unsurprisingly, has a substantial impact on the mental well-being of adults, with over 40% of US adult residents expecting that they or an immediate family member could become a victim of gun violence [24-28]. In particular, school staff must now contend with gun violence or the possibility of gun violence in their schools. This burden also requires them to attend to the mental health needs of their students, and for some educators, reckoning with exposure to gun violence or the possibility of such exposure may take a significant emotional and physical toll. The National Education Association has referred to this phenomenon as “secondary traumatic stress” [29]; however, there is need for research that more clearly establishes the strength of the relationships among students and school staff who have either been exposed to gun violence or are contending with the anticipation of gun violence, and their mental health and well-being.

### Adverse Childhood Experiences

The scientific literature conceptualizes ACEs as stressful or traumatic events that exert harmful impacts on the healthy development of children through adolescence and into adulthood [30]. Although historically research on ACEs has focused on child abuse and maltreatment, ACEs are now understood to include a wider range of traumatic events, including youth experiences with the juvenile justice system, household mental illness, family members who have been incarcerated, and childhood exposure to gun violence [17,18,30-33]. The cumulative and long-term influence of ACEs on multiple harmful and injurious behaviors has been well established [18]. Research has confirmed that ACEs increase a person’s risk of poor mental health, illicit drug use, self-harm behaviors, and premature mortality [18,19]. The impact of the toxic stress typically associated with ACEs on a child’s brain development is also well-documented [20]. More recently, research has established a clear relationship between an increased number of ACEs and poor academic outcomes in children [17]. Thus, it is quite possible that those students who have a higher number of ACEs may experience poorer mental health and educational outcomes following exposure to intentional school shootings and school safety strategies implemented in response to the anticipation of such violence, in comparison with their peers who have experienced very few or any ACEs. At the same time, there is an emerging body of research on positive childhood experiences (PCEs), which include feeling a sense of belonging, having meaningful and positive relationships, and that collectively may mitigate the impacts of ACEs on child outcomes [34]. This work will seek to understand and incorporate the role of PCEs on youth mental health, well-being, perceived safety, and learning.

### Exposure to School Violence

Previous research has shown that exposure to different forms of violence are ACEs in and of themselves [32,33]. Similar to other ACEs, the impact of exposure to violence on children’s short- and long-term health outcomes is significant. For example, research has demonstrated that children and adolescents who have recently witnessed violence are more likely to experience symptoms of trauma, including the onset of posttraumatic stress [15,35]. There are multiple studies confirming that exposure to violence during childhood is associated with poor chronic health outcomes and poor mental health, as well as a significant increase in the likelihood that young people who are exposed to violence will perpetrate violent crime themselves [33,36-38]. Moreover, there is a substantial body of research on school violence that has linked youth exposure to violence, specifically in school settings with multiple poor health and learning outcomes, such as decreased motivation to learn and lower classroom engagement [17,39]. Not surprisingly, exposure to violence also influences health outcomes during adulthood (including increasing one’s likelihood for depression and other poor mental health outcomes, sleep quality, chronic disease, and substance use) [30,40-42]. The effect of exposure specifically to intentional school gun violence on youth and adult mental health outcomes and youth educational outcomes, however, has not been comprehensively documented.

### Influence of School Climate

A robust set of research confirms that children who feel dissatisfied with, disconnected from, or unsafe within their school environment are more likely to experience poor health and learning outcomes [39,43-45]. If young people feel unsafe or threatened, their ability to focus and perform academically is drastically reduced [39]. Furthermore, perceptions of safety at school and prior exposure to violence have been, and continue to be, highly correlated with engagement in many risk behaviors [43-45]. Conversely, a nurturing school climate that fosters cohesion and student satisfaction with their academic learning has been shown to offset high-risk behaviors [45]. However, there has been an uptick in intentional school gun violence over the past few years [8,9]. In addition to likely influencing a school’s social and emotional climate [46], exposure to such violence has substantial implications for a school’s physical environment and has prompted schools, both those that have experienced intentional gun violence and those that have not, to increase school violence preparedness [10-15]. Such school safety tactics include target hardening efforts, zero-tolerance policies, presence of armed school resource officers and armed teachers, and emergency preparedness programs [10-14,47-49]. While there are a number of strategies available to schools, there is very limited empirical evidence as to their effectiveness. Of the evidence that exists, certain security efforts have been shown to be ineffective at deterring violence and may even have a criminalizing effect on a school’s climate [10,12]. Given this collective body of work, it is reasonable to hypothesize that these school safety strategies may contribute to poor mental health and distress among students and staff. There is, however, very limited empirical work that has evaluated this relationship [29,50].

### Purpose

Poor mental health symptoms and ACEs among the youth predict extensive co-occurring adverse outcomes, including increases in long-term risk for chronic disease and injury, impaired child development, and poor academic outcomes [17-20]. At the same time, the anticipation of school shootings has fueled school safety and security interventions that may inadvertently increase anxiety, depression, and other indicators of poor mental well-being among students and staff alike [29,50]. Despite this, the association between exposure to existing school safety interventions and student mental health outcomes, particularly beginning in early adolescence, is not clear. It is also unclear whether, how, and to what extent the strength of these associations might differ among students and staff members from those schools that have more recently experienced a school shooting, those that have experienced a school shooting less recently, and those that have never directly experienced a school shooting but are nonetheless indirectly aware of these tragic events via media and other networks. Finally, we do not know whether and to what extent having a prior history of ACEs moderates the relationships among exposure to school shootings, exposure to school safety strategies, and student outcomes (eg, perceptions of school safety and academic achievement). The role of PCEs is also unknown. This study therefore seeks to fill these critical research gaps by answering the following questions:

1. Is there a significant difference in the prevalence of mental health outcomes, perceived school safety, and academic engagement between early adolescent and adolescent students (grades 6-12) at public secondary schools that have experienced a school shooting and those that have not?
2. Are existing interventions to promote school safety and security associated with poor mental health outcomes among students and school staff members?
3. Does the strength of this association between school safety interventions and mental health outcomes differ among students and teachers who have experienced an intentional school shooting versus students and teachers who never experienced a school shooting? Furthermore, are these associations moderated by student ACE history?

## Methods

### Sampling Frame

#### Study Design

This is an observational study that will involve collecting cross-sectional survey data from a national sample of students, teachers, and principals from 12 secondary public schools across the United States. Our sample includes participants from 6 randomly selected exposure schools: secondary schools that have recently experienced an intentional school shooting (<2 years ago) and secondary schools that have experienced an intentional school shooting less recently (>2 years ago). Of the schools in our dataset that meet these criteria, we are randomly selecting schools to participate in our study from the group of eligible schools. If a school does not express interest in participating, we are replacing that school with another eligible school in our dataset. We are then matching our exposure schools to 6 randomly selected nonexposure schools: 6 secondary public schools that have never experienced a school shooting. We are matching the nonexposure schools to exposure schools on the following key variables: state, urban or nonurban status, and school level (ie, middle or high school).

#### Exposure School Selection

Our research team is identifying all possible intentional school shooting cases via a comprehensive review of multiple national school shooting databases, including the K-12 School Shooting database, the Everytown for Gun Safety database on gunfire on school grounds, and the Washington Post database on school shootings, to identify all schools that have experienced intentional gunfire on campus during school hours [51]. These data document all incidents in which a gun is fired or a bullet hits school property for any reason, regardless of the number of victims, time, or day of the week. Our research team is then coding this list of incidents to identify cases that meet the criteria for intentional school shooting at a secondary (ie, middle or high school level) public school—all cases of intentional gunfire on school property, occurring during school hours (including 1 hour before official school activities begin and 1 hour after all official school activities end), beginning January 1, 2015—to ensure we have enough schools in our sampling frame that meet our aforementioned criteria. We are excluding accidental discharges and attempted suicides where no other person was targeted or shot. We are also excluding any incidents taking place on a school bus and on school property that are noncontiguous with the school’s primary campus. Furthermore, we are cross-referencing our list of incidents with other publicly available data sources [40] to ensure that no incidents meeting our criteria are missed. Two members of our research team are then independently coding this list of incidents to identify the cases that meet our proposed study’s criteria for “intentional school shooting”; a third member of our study team is reviewing any potential discrepancies in coding efforts. The final list of exposure schools will then be divided into 2 based on recentness, that is, schools where the incident happened between January 1, 2015, and December 31, 2021, and schools where the incident happened between January 1, 2022, to December 31, 2023. In total, 3 schools will be randomly selected from each list. In case a school declines to participate, we will move to the next randomly selected school. Nonexposure schools will be matched to the final exposure schools. In case a school declines to participate, we will move to the next randomly selected school.

#### Recentness

To operationalize the “recentness” of a school shooting event, we considered the existing literature on posttraumatic stress. The evidence shows that the onset of posttraumatic stress disorder (PTSD) symptoms can vary drastically (eg, symptoms may appear within 3 months of the traumatic experience or be delayed by months or even years) [52-54]. A 2-year cutoff, therefore, allows us to assess the difference between a more immediate impact versus a longer-term impact on youth mental health outcomes. Similarly, we expect shifts in perceived school safety, school safety strategies, and the implementation of other related school initiatives and programs to vary considerably in the aftermath of a school shooting incident. The 2-year cutoff will allow us to compare schools in the midst of that “flux” to those schools that have had some time to adapt their practices and policies following exposure to such violence.

#### Nonexposure School Selection

To select the nonexposure schools, we are randomly selecting 6 schools from a database of all public secondary schools in the United States [55], matching for the aforementioned variables (ie, state, urban or nonurban, and school level).

#### Students

Within each participating school, all students between grades 6 to 12 are eligible for and invited to participate in this study. To encourage study participation, we are implementing reasonable incentives for participants, keeping the survey as brief as possible, communicating study expectations clearly and consistently, engaging thoughtfully with each school’s leadership team during the recruitment process, and using other best practices [56,57]. Accounting for attrition and individuals choosing not to participate, we expect a 15% to 20% response rate (which is consistent with other research that has involved collecting survey data directly from students, teachers, and principals) [58,59], and therefore, anticipate sampling between 1000 to 1200 students across the 12 participating schools.

#### Teachers

Within each participating school, all teachers are eligible for and invited to participate in this study. Multiple efforts are being made to maximize participant recruitment and retention. We anticipate a minimum of 60 teachers and upward of 120 teachers (5-10 teachers per school, on average) to participate in this proposed study.

#### Principals

The current principal of each school is eligible and being invited to participate in this study.

### Participant Recruitment

#### Recruitment Process

In mapping out the study’s recruitment process, the project team has drawn on existing best practices to ensure the study’s success. First, the project team defined a clear sampling frame for school recruitment to ensure that the 12 schools being recruited fall within the purview and meet the purpose of the study’s aims. We are also providing reasonable and feasibly budgeted incentives for all participating schools (specifically, a US $250 gift certificate to Amazon) as a small way to appropriately recognize each school’s time and commitment to this study effort. To ensure that this effort takes only minimal time away from the existing school day and does not detract from the various responsibilities of all participants, the project team has worked to ensure that each aspect of the data collection process is as brief and efficient as possible. All of these details are being conveyed to the potential participating schools during the recruitment process.

#### Principal and Teacher Recruitment

In line with best practices, the principal at each potential participating school within our sampling frame is being approached directly through a written letter (sent via email). After 2 weeks, and if there is no response, the co–principal investigators (co-PIs) of the study are following up through a telephone call. The co-PIs are then meeting with the principal via Zoom (Zoom Video Communications, Inc) at their convenience to engage with them and describe the processes and expectations of the study for the school’s participation, as well as the corresponding risks and benefits, and provide opportunity for the principal to ask questions (with additional opportunity for further conversations, as needed). If the principal agrees to support the study effort, the co-PIs are working closely with each principal to then recruit the school’s teachers and students for participation in the study. The project team’s previous research experience conducting this kind of research suggests that this engagement approach is beneficial for participant recruitment and aims to ensure participant comfort and transparency with all aspects of the study protocol.

To maximize principal and teacher participation, the co-PIs are organizing an optional 30-minute recruitment meeting initially via Zoom at each school (to be scheduled, with the principal’s support, during the school’s professional development hours). During this time, the co-PIs are presenting an overview of the study’s purpose and proposed methods, highlight any relevant risks and benefits (including provision of incentives), describing the informed consent process, and providing an opportunity for the principal and teachers to ask questions about the study itself.

It should be emphasized that participation in this study is relatively brief and will involve the completion of 1 web-based survey. The informed consent form will be included at the beginning of the survey. However, as noted above, part of the recruitment meeting will involve the co-PIs answering any questions about the study and its corresponding informed consent process.

#### Student Recruitment

Similarly, to maximize student participation, the co-PIs are organizing an optional 30-minute recruitment meeting via Zoom for the students and their parents at each school. During this time, the co-PIs will present an overview of the study’s purpose and proposed methods, highlight any relevant risks and benefits (including provision of incentives), describe the informed consent process, and provide an opportunity for the students and parents to ask questions about the study itself. Again, during this time we are emphasizing that participation is relatively brief and will involve the completion of 1 web-based survey. The informed consent form is also being included at the beginning of the survey (and for students older than 12 years, this will also include an assent form). As noted earlier, part of the recruitment meeting will involve the co-PIs answering any questions about the study and its corresponding informed consent process.

### Measures

#### Data Collection Overview

This study involves collecting cross-sectional survey data from students, teachers, and principals from 12 public secondary schools across the United States. Publicly available secondary data about each school will also be collected and used. All participants will complete the survey on the web.

#### Demographics

Data on sex, race, age, grade-level, and presence or not at the time of the school shooting incident for students in the exposure school will be provided by each student at the beginning of the survey. Self-report data on teacher and principal demographics (eg, their length of time working at the school) will also be collected at the beginning of the teacher and principal surveys, respectively.

#### Mental Health and Well-Being

Among the participating students in each school, self-report data on mental health and well-being will be assessed via a survey that comprises items from the following: (1) the Patient Health Questionnaire (PHQ)–A [60], a validated and widely used survey assessing mental health and symptoms of depression among the youth; (2) the Child and Adolescent Trauma Screen [61], which has been validated for use by young people aged 7 to 17 years; and (3) the World Health Organization Well-Being Index (WHO-5) [62], which has been validated for use by individuals 9 years and older. The PHQ-A and Child and Adolescent Trauma Screen items are multiple-choice with 4 response options, and the WHO-5 items are rated on a 6-point Likert scale [60-62].

Among the participating teachers in each school, self-report survey data on mental health and well-being will be assessed via items from the following: (1) the PHQ-9 [63], a validated scale assessing mental well-being and symptoms of depression among adults; (2) the Generalized Anxiety Disorder-7 scale [64], which has also been validated and assesses the most common anxiety disorders among adults; and (3) the WHO-5 [62]. The PHQ-9 and Generalized Anxiety Disorder-7 items are multiple-choice with 4 response options, and the WHO-5 items are rated on a 6-point Likert scale [62-64].

#### ACE and PCE Prevalence

Each student will provide self-report data on ACE and PCE prevalence. The ACE items were adapted in previous research [17] from a validated tool [19]. Building upon additional research that has argued that children experience a greater range of adversity than most ACE assessments currently capture [31,33], additional items will be included. The survey will, therefore, assess the following: bullying, residential instability, meeting of basic needs, divorced parents, family member with poor mental health, family member engaging in substance use, domestic violence, incarcerated parent, community violence, exposure to gun violence, and exposure to violence in school. Each item will be multiple-choice, with the following response options: “yes,” “no,” or “prefer not to answer,” and these data will comprise an ACE prevalence score that denotes how many ACEs each student has experienced. PCE items were similarly adapted in previous research [34].

#### Perceptions of School Safety

The “Safe and Responsive Schools” Safe School Survey [65] is a validated tool designed to be administered to students and school staff. This instrument assesses six critical constructs, including perceptions of school climate, school safety, and belongingness. In line with previous research on school violence and in an effort to comprehensively assess perceptions of school safety, this tool seeks to capture both minor conflicts (eg, arguments among students) as well as more significant forms of disruption (eg, weapon possession on school grounds). Each student survey will include items from the Safe School Survey, which has a 5-point Likert scale response option [65]. Each teacher survey will include items from the Safe School Survey designed for school staff, which has a 5-point Likert scale response option [65]. Furthermore, each principal survey will also include items from the Safe School Survey designed for school staff [65].

#### Educational Outcomes

Among the participating students in each school, self-report survey data on academic engagement will be assessed via 4 items stemming from the Student Engagement Instrument [66,67]. The Student Engagement Instrument is a survey that assesses extrinsic motivation to learn, future goals and aspirations, class participation, teacher-student relationships, and perceived peer support for learning [66,67]. We will also include 1 multiple-choice item that has been adapted from the Centers for Disease Control and Prevention’s Youth Risk Behavioral Surveillance System that asks students to describe their academic grades in schools over the past 12 months [68].

#### School Safety and Security

Data on school safety and security strategies for each participating school will be collected via 2 sources. First, principals from all participating schools will be surveyed on their knowledge of current school safety tactics and policies. Data on school safety and security indicators under the following seven categories will be assessed: (1) external target hardening efforts (eg, monitored school entry doors and signs indicating the school is a “Gun Free School Zone”); (2) internal target hardening efforts (eg, metal detectors and security cameras); (3) student or staff monitoring (eg, threat assessment team and zero-tolerance policies for weapons); (4) emergency procedures or drills (eg, written active shooter plan); (5) emergency notification technologies (eg, anonymous thread reporting systems and panic buttons); (6) medical support (eg, full or part-time school nurse on campus); and (7) school security staff (eg, law enforcement officers or police and teachers and other personnel armed with guns). Each multiple-choice item will include the following response options: “Yes, this policy/practice is active now at my school,” “No, this policy/practice is not active at my school,” or “Don’t know/No answer.” It should be noted that several of these survey items were adapted from the School Survey on Crime and Safety that is administered via the National Center for Education Statistics to a nationally representative sample of schools to assess whether individual schools are or are not implementing a range of school safety and security interventions [69]. First, for each school, a summative score will be generated that accounts for the number of policies or practices that each principal responded with “yes.” Second, and especially if the principals do not necessarily have the time and capacity to review the safety details via a survey, we will review each school’s current school safety plan, which is a comprehensive document developed by the school stakeholders that details preparation and response protocols to various school emergencies (ranging from bullying prevention efforts to fire safety drills and active shooter scenarios). A thorough review of each school’s safety plan will allow our team to validate the data collected about current school safety strategies and also fill in any missing data. Should there be any discrepancy between the data provided by the principal and the protocols detailed in the school safety plan, the study team will defer to the data provided via the school safety plan.

#### Active Shooter Drills

Given the ubiquity specifically of active shooter drill implementation across secondary schools in the United States [15], we will identify each school’s active safety drill protocol via the school’s safety plan in our school recruitment effort for both exposure and nonexposure schools, and subsequently in our analyses. Specifically, we will identify whether our schools are currently implementing the Standard Response Protocol [70], which is the most widely implemented active shooter drill protocol in schools across the United States and involves high student-teacher preparedness and processing, versus other types of drill protocols [71] that may involve low student-teacher preparedness, a surprise element to the protocol, and more realistic portrayals of violence built into the drill. This will help us to identify and subsequently classify schools in our sample with two varying types of active shooter drill implementation.

#### Independent Covariates

Multiple independent covariates will also be collected via data sources like the National Center for Education Statistics and the US Census and adjusted for in conditional logistic regression models [72,73]. These covariates will include the length of time since the school experienced a shooting, demographics (eg, urbanicity, school level, and type of school), and school district characteristics. In addition, secondary data on education access, equity, and school discipline will be directly downloaded from the Civil Rights Data Collection website [74]. Using these recent data on each participating school, the study team will create a “school discipline” summative score that will comprise the following indicators (accounting for school population size and reflecting the number per academic year): attendance rate, number of in-school suspensions, out-of-school suspensions, students referred to law enforcement, students disciplined for harassment or bullying, and school-related arrests. Propensity score match and control statistical methods will be used to ensure rigor in association validity, controlling for these characteristics comprehensively.

### Data Collection Procedures

#### Students

Participating students in each school will complete 1 web-based survey administered on the web via Qualtrics (Qualtrics, LLC) [75], which is an web-based survey platform. This survey will take approximately 10 to 12 minutes to complete.

#### Teachers

Participating teachers will complete 1 on the web, also via Qualtrics, that will take approximately 10 to 12 minutes to complete.

#### Principals

Participating principals will complete one 10- to 12-minute survey, again via Qualtrics. As part of the principal survey, we will be asking them to provide a copy of their school’s current school safety plan.

### Ethical Considerations

#### Protection for Human Subjects

This study protocol (protocol #21-149) underwent a full board review and has been approved by the institutional review board (IRB) at Teachers College, Columbia University. Details on data confidentiality and privacy protections, informed consent descriptions, the study’s inclusion criteria, and participant compensation are noted in the following sections.

#### Data Privacy and Confidentiality Protection

Survey data collected from students, teachers, and school staff will be confidential. All participants will be associated with a specific school. However, no identifying names or any other identifiers will be used at any point in the study. Each participant will be identified solely via an alphanumeric code during the data collection and organization process. This will be used solely for matching the survey data to the appropriate school for analyses. No information about any individual student or staff will ever be reported or shared. Furthermore, all survey data will be reported in aggregate.

#### Informed Consent Descriptions

As described in the participant recruitment section, all potential participants will engage in a thoughtful informed consent process. Parents and guardians of all students will have the opportunity to provide consent, if they choose, for their child to participate in this study. Similarly, the potential teacher and principal participants will also complete a consent form before participation. These consent forms, all also approved by the Teachers College, Columbia University IRB, review in clear language the purpose of the proposed study, what each participant will be asked to do if they agree to participate, any potential risks, discomforts, and benefits each participant might expect from taking part in the study, details about the study’s confidentiality, how the results will be used, and the co-PIs’ contact information should any questions arise during this process. For those students whose parents provide consent for them to participate, they then will have the opportunity to complete an assent form before participating in the study’s survey. The assent form for the students similarly and clearly describes the purpose of the research, what the student can expect during the study itself, any consequences associated with participating in this study, how we will take care to protect their data privacy and confidentiality, the co-PIs’ contact information should any questions arise, and also a statement underscoring that participation is completely voluntary and that the student does not have to participate.

#### Inclusion

The literature that has been synthesized for this study elucidates the critical role that members of minority groups, members of both sexes, and children all play in a study like this. Thus, members from all 3 groups are included.

#### Compensation

Each participating school will receive a US $250 gift card to Amazon for the school to put toward books or other school supplies. Following participation in the study, the study’s co-PIs will send the electronic gift card directly to the participating school’s principal.

### Data Organization and Treatment

#### Survey Data Entry

All survey data collected in this study are being recorded initially in Excel (Microsoft Corp) for organization and management and then subsequently read into SPSS (version 29.0; IBM Corp) for analysis [76]. The study team maintains a codebook; variables will be recoded where needed and summative scores will be computed.

#### Document Review

Each school safety plan will be reviewed and coded independently by 2 members of our research team to determine if each of the school safety strategies and tactics of interest are being implemented. These data will also be recorded initially in Microsoft Excel for organization and management and then subsequently read into SPSS for analysis.

#### Missing Data

Consistent with previous research, we estimate the majority of survey items to have <10% missing data and no more than 19% missing survey data on any given item [58]. However, should higher levels of missing data occur, multiple imputation methods will be used [77].

### Data Analysis Plan

The data collected from the students, teachers, and principals across all 12 participating schools will be analyzed and will fully respond to each of the research questions. Descriptive statistics will be calculated to summarize student and teacher participant characteristics within each school and also describe each school’s safety and security strategies. Analyses of covariance will subsequently be used to identify potential significant differences in the prevalence of mental health and well-being, perceptions of school safety, educational outcomes, and ACEs among students in schools that have more recently experienced an intentional school shooting, schools that have experienced an intentional school shooting less recently, and schools that have never experienced a shooting, while controlling for key covariates.

ANOVA will also be used to identify potential significant differences in the prevalence of mental health and perceptions of school safety among teachers in schools that have more recently experienced an intentional school shooting, schools that have experienced an intentional school shooting less recently, and schools that have never experienced an intentional school shooting. We used G^*^Power (version 3.1; Heinrich Heine University Düsseldorf) to run power analyses in anticipation of our proposed analyses, assuming an α level of .05 and a power of 0.80. In the case of our proposed student analyses, a very small minimum effect size will need to be detected to meet our power requirements and given our anticipated minimum sample size (exposure schools: N=500-600, nonexposure schools: N=500-600). In the case of our teacher analyses, a medium effect size will need to be detected and given our anticipated minimum sample size (exposure schools: N=30-60, nonexposure schools: N=30-60). On the basis of the average enrollment across secondary public schools in the United States, we should be able to reach the minimum sample size from 12 schools.

Multiple regression analyses will then be used to determine if the strength of the association between school safety interventions and mental health outcomes differs among students and school staff in schools with varying levels of trauma exposure. Mediated moderation analyses will evaluate the role of ACEs on the relationships between exposure to an intentional school shooting, exposure to school safety strategies, and student outcomes (ie, mental health and well-being, perceptions of school safety, and educational outcomes) [78]. Again, power analyses revealed that for our student-level analyses and given an α level of .05 and a power of 0.80, a small effect size will need to be detected to meet our power and sample size requirements. In the case of our proposed teacher analyses, a medium effect size will need to be detected. It should be noted that these values reflect conservative estimates with regard to student and staff recruitment. Thus, we are confident that we will achieve reasonable power to fully answer all of our questions and achieve the specific aims of the study.

## Results

IRB approval for this research project was obtained and the study subsequently began its recruitment and data collection phase in January 2024. Data collection is currently ongoing, and the expected completion date is January 2025. As of May 2024, we have recruited and administered the surveys at 3 secondary public schools. The full data analysis is expected to be completed by August 2025.

## Discussion

### Overview

Poor mental health symptoms and ACEs among the youth predict extensive co-occurring adverse outcomes, including increases in long-term risk for chronic disease and injury, impaired child development, and poor academic outcomes [17-20]. At the same time, the anticipation of intentional gun violence has fueled school safety and security interventions that may increase anxiety, depression, and other indicators of poor mental well-being among students and staff alike [29,50]. Despite this, the association between exposure to existing school safety interventions and student mental health outcomes is not clear. It is also unclear whether and how the strength of these associations might differ among students and staff from those schools that have more recently experienced gun violence, those that have experienced intentional gun violence less recently, and those that have never directly experienced intentional gun violence but are indirectly aware of these tragic events via media and other networks. Finally, we do not know whether and to what extent having a prior history of ACEs moderates the relationships among exposure to intentional gun violence at school, exposure to school safety strategies, and student outcomes.

There is no research that has documented the burden of mental health problems on cohorts of students subsequent to actual, as well as anticipated, school violence. Although the likelihood of an intentional school shooting occurring in a secondary school is relatively low in comparison with other forms of gun violence that occur in our communities [79], the anticipation of such violence in response to the violence experienced over the past decade has led schools to actively consider how best to keep their students and staff safe. Contributing to this movement is the fact that, although there are many schools that have never directly experienced a school shooting, students and staff nonetheless are indirectly aware of these tragic events via news coverage, social media, and other communication channels.

At the same time, there is a paucity of available evidence on the effectiveness of many school safety strategies and, importantly, their impact on student and school staff outcomes. Indeed, there are growing concerns that certain safety strategies (eg, active shooter drills) are potentially traumatic. Furthermore, exposing children, in particular, to such traumatic events may have significant implications for health and learning outcomes in both the short- and long-term. It is also unclear if exposure to a school shooting on student outcomes (ie, mental health and well-being, perceptions of school safety, and academic outcomes) might be more pronounced among students with a history of ACEs.

### Potential Study Challenges, Limitations, and Strategies to Address Them

We acknowledge the potential challenges this study presents, and we have identified approaches to mitigate specific concerns that may arise in the implementation of the study. The primary challenge is that this study requires voluntary participation of 12 schools, their students, and staff. As noted earlier and to encourage participation, every effort is being made to minimize the time burden associated with participation in this study, prioritize confidentiality of data, and create an inclusive study environment by ensuring that all participants have regular opportunities to ask questions and express concerns throughout the study process. We will also be providing incentives to all participating schools to recognize their time spent on this study.

Other potential risks include emotional responses that may emerge during the data collection process. It is important to underscore that great care will be taken to ensure that the proposed data collection effort takes place in a manner that is comfortable and safe for all participants. It should be noted that our study will not be collecting data on self-harm or suicidality. In addition, the representativeness of our school sample may pose to be a limitation. Indeed, there is such variation in school size, urbanicity, variation by state, and budget, that the schools themselves may not be sufficiently representative of all possible schools meeting our criteria. However, as noted earlier, our sample has sufficient power and we have ensured representation to the best of our ability across geographic regions. Finally, there is the possibility of missing survey data; however, we will be using multiple data sources where possible and multiple imputation methods will be used to address the problem of missing data during the data analysis process.

### Conclusions

Despite the potential challenges that we may encounter in conducting this study, we anticipate the results of this work will fill a significant gap in the literature on the impact of exposure to gun violence as an ACE. Ultimately, we believe the results of this study could hold the promise of not only contributing to understanding the relationship of exposure to gun violence to a range of youth, school, and health outcomes, but also providing schools with more data-driven evidence to more effectively inform their firearm violence prevention practices, the allocation of mental health resources, and other interventions, as they simultaneously consider how best to cultivate a positive school climate and attend to the mental health and well-being of their students and staff.

## Abbreviations

ACE: adverse childhood experience
co-PI: co–principal investigator
IRB: institutional review board
PCE: positive childhood experience
PHQ: Patient Health Questionnaire
WHO-5: World Health Organization Well-Being Index

## Data Availability

The datasets generated during and analyzed during this study are not publicly available but are available from the corresponding author on reasonable request.
